# Surface morphology, optical properties and conductivity changes of poly(3,4-ethylenedioxythiophene):poly(styrenesulfonate) by using additives^☆^

**DOI:** 10.1016/j.tsf.2013.03.124

**Published:** 2013-06-01

**Authors:** Jacek Gasiorowski, Reghu Menon, Kurt Hingerl, Marko Dachev, Niyazi Serdar Sariciftci

**Affiliations:** a Linz Institute for Organic Solar Cells (LIOS), Physical Chemistry, Johannes Kepler University of Linz, Austria; b Department of Physics, Indian Institute of Science, Bangalore 560012, India; c Zentrum fur Oberflachen-und Nanoanalytik, Johannes Kepler University of Linz, Austria

**Keywords:** Optical properties, Conductivity, Surface morphology, Conducting polymers, Spectroscopic ellipsometry, Organic electronics

## Abstract

The optical properties and electrical conductivity of highly conducting poly(3,4-ethylenedioxythiophene) (PEDOT) doped with poly(styrenesulfonate) (PSS) are reported as a function of the processing additive conditions. The addition of dimethyl sulfoxide (DMSO) increases the conductivity and modifies the dielectric response as observed from the ellipsometric studies. Also the surface roughness and morphology change with the composition of PEDOT:PSS:DMSO and film deposition conditions. The real part of the dielectric function becomes negative in highly conducting samples, indicating the presence of delocalized charge carriers. The real and imaginary parts of the refractive index were determined as a function of wavelength. The results are consistent with the increase in conductivity upon the addition of DMSO.

## Highlights

• PEDOT:PSS was mixed with dimethyl sulfoxide.
• The conductivity increase due to the coil extention is correlated with morphology.
• Conductivity change correlates with morphology and extention of compact-coils.
• The carrier delocalization is correlated with the dielectric function.

## 1 Introduction

**Fig. 1:**
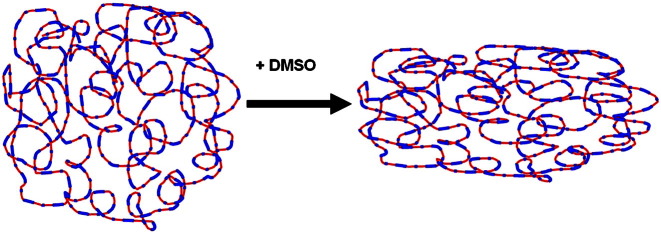
The PSS (red) is sparingly covered by short PEDOT segments (blue) creating spherical nanostructure. After adding DMSO the coil structure elongates into an ellipsoidal form.

The highly conducting poly(3,4-ethylenedioxythiophene):poly(styrenesulfonate) PEDOT:PSS (with a brand name PH1000, Clevios Heraeus Co.) has the potential to be used as a viable alternative to the usual transparent conducting oxides like indium-tin oxide (ITO), doped zinc oxide, etc. in electronics [1–4]. Although the optical transparency of the earlier versions of PEDOT:PSS is rather close to that of ITO, the relatively high resistance has remained an obstacle in these optoelectronic applications. Earlier studies have shown that chemical processing of PEDOT:PSS plays a major role in changing its morphology and conductivity [5–11]. By mixing PEDOT:PSS with various solvents like dimethyl sulfoxide (DMSO) and also by annealing at higher temperatures the conductivity varies by several orders of magnitude [12,13]. Since PEDOT:PSS is a rather complex system, as shown in the schematic diagram in Fig. 1, the PEDOT rich parts of the chains tend to diffuse more towards the core and the PSS rich portions form a shell like structure in these gel particles of wide range of sizes from micro to nano scale [14,15]. In usual aqueous PEDOT:PSS solution the chains tend to form compact coils that pack randomly upon drying in solid state films. In this case, the nanomorphology always plays a significant role in the bulk charge transport, hence a wide range of values of surface resistance in transparent electrode application has been reported [16]. However, it has been observed from small angle X-ray scattering and charge transport studies that in the presence of additives the compact coils transform to more elongated ones so that the localized electronic states get partially delocalized to enhance the transport mobility of charge carriers [17,18]. In this case the barriers will be lowered in the elongated conformation of chains facilitating charge transport. The effect of these morphological modifications can be observed especially in the low temperature charge transport properties and frequency dependent conductivity.

In this work a systematic study has been carried out to investigate the effect of the addition of DMSO on morphology, optical absorption and conductivity of PEDOT:PSS thin films. For the spin-coated layers, the thickness and surface roughness have been determined from atomic force microscope (AFM) measurements for various DMSO concentrations. The optical properties of the pristine PEDOT:PSS as well as mixed with DMSO were studied using spectroscopic ellipsometry and real and imaginary part of their dielectric functions were determined. Using the complex dielectric function, the absorption coefficient, and the complex refractive index were calculated. The results were compared with a less conducting form of PEDOT:PSS (brand name PH510, Clevios Heraeus Co.) for a better understanding of the influence of free charge carriers on their optical properties. Finally the conductivity values have been measured by using four probe technique in all PEDOT:PSS (PH1000) samples. The effect of thickness and surface roughness of the samples on the optical and electrical properties is also investigated in this study.

## 2 Experiment

In this work, an aqueous solution of PEDOT:PSS (brand names PH1000 and PH510, Clevios Heraeus Co.) was used. The pristine PEDOT:PSS (PH1000 and PH510) solutions were compared with solutions with 5%, 10%, 15% and 20% v/v concentrations of dimethyl sulfoxide (≥ 99.5%, Sigma Aldrich) in PEDOT:PSS:DMSO solution. These solutions were deposited by spin coating using P6700 spincoater (Speciality Coating Systems, Co.) on a 15 × 15 mm^2^ glass substrate from Menzel Gläser. The glass substrates were precleaned by sequential sonication in acetone (99.8%, NORMAPUR), isopropanol (99.9%, NORMAPUR) and de-ionized water. Before spin coating, the solutions were filtered using Whatman Puradisc AS25 filters. For the thickness variation two different spin coating programs were used, allowing us to obtain two thicknesses — approx. 50 nm (high rpm — 2000 rpm, 1 s + 4000 rpm, 1 s + 6000 rpm, 60 s) and 120 nm (low rpm — 1500 rpm, 40 s + 2000 rpm, 20 s). The thickness and surface morphology of the films were characterized by atomic force microscope (Digital Instruments Dimension 3100, Veeco Metrology group) working in the tapping mode. Ellipsometric characterization was done using Woollam M-2000 (rotating compensator) ellipsometer which spans an energy range of 0.73 to 6.5 eV. The linear four probe technique was applied to measure the conductivity by using Keithley 2400 sourcemeter.

## 3 Results and discussion

**Fig. 2:**
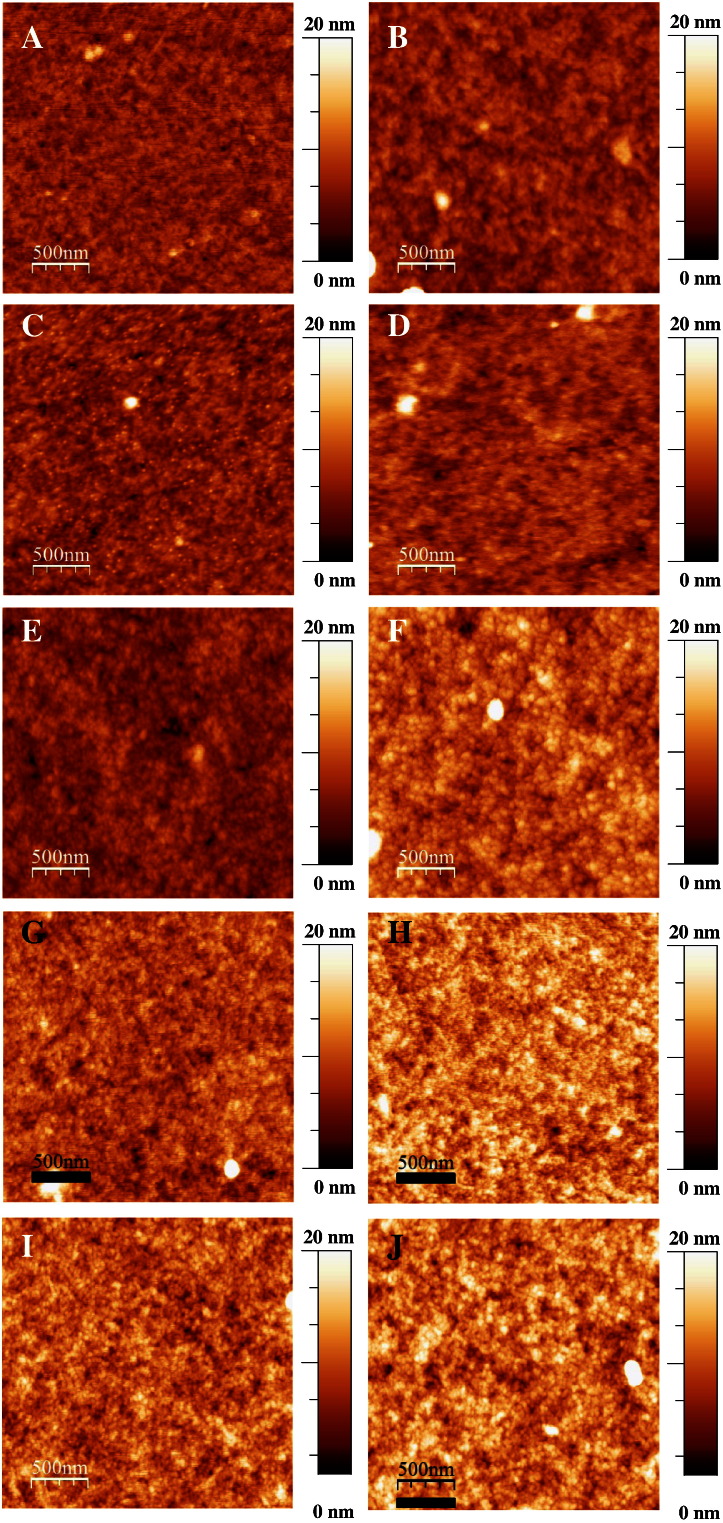
AFM characterization of surface morphology of thin and thick film of PEDOT:PSS (A and B) and PEDOT:PSS mixed with 5% (C and D), 10% (E and F), 15% (G and H) and 20% (I and J) v/v DMSO.

**Fig. 3:**
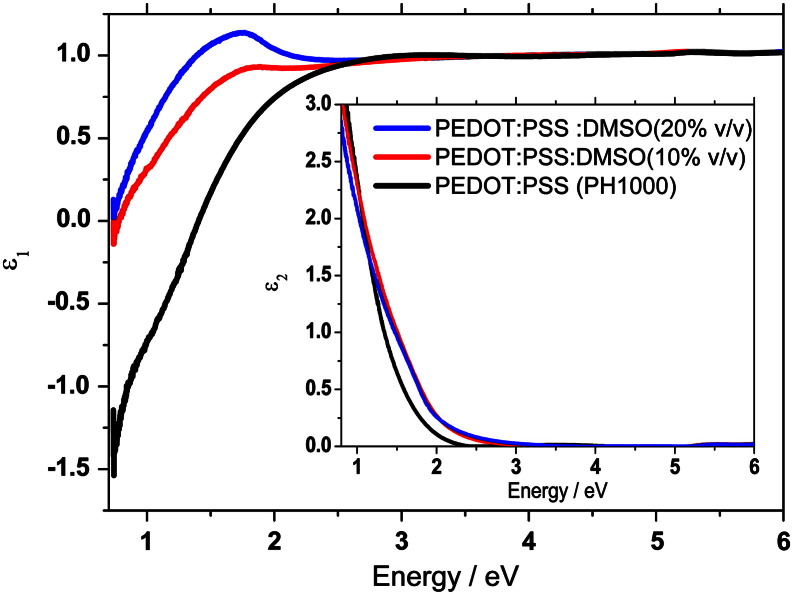
Real part of the dielectric function of the pristine PEDOT:PSS (PH1000) (black) and PEDOT:PSS (PH1000) mixed with 10% (red) and 20% (blue) v/v DMSO.

**Fig. 4:**
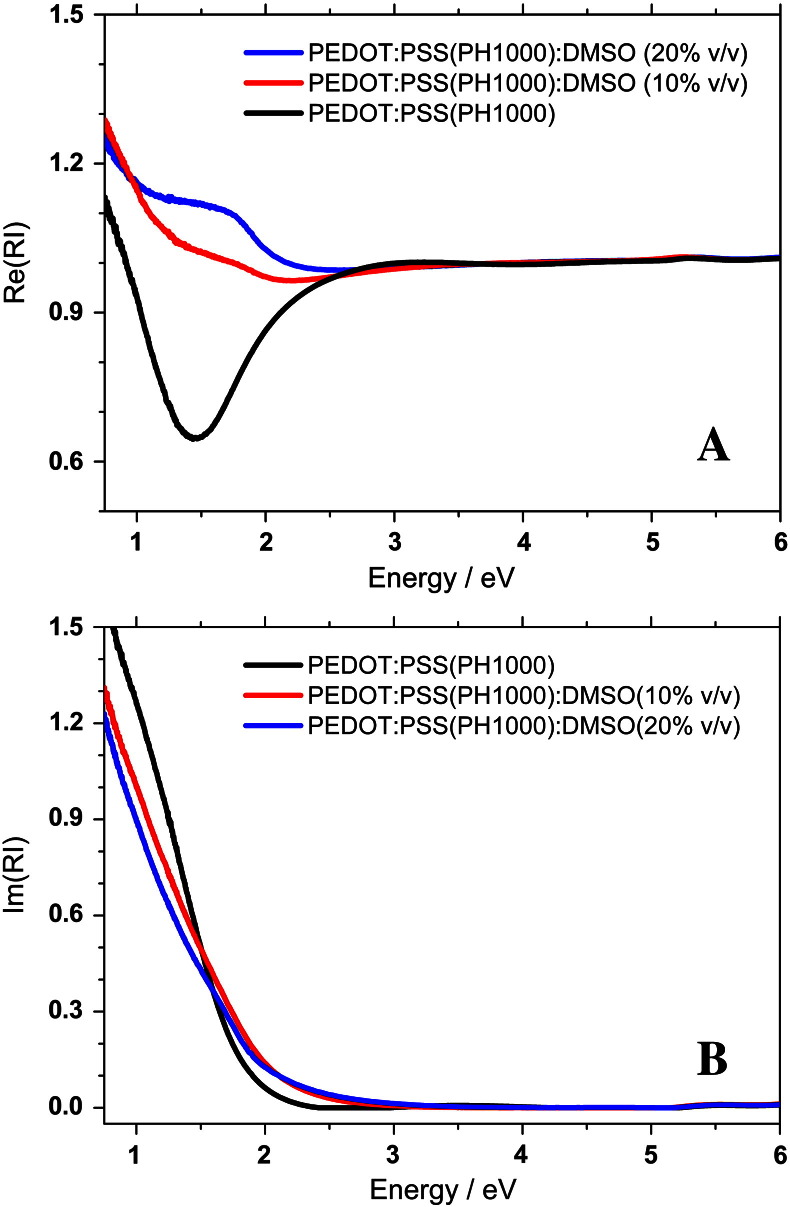
The real (A) and imaginary (B) parts of the refractive index of the pristine PEDOT:PSS (PH1000) (black) and PEDOT:PSS (PH1000) mixed with 10% (red) and 20% (blue) v/v DMSO.

PEDOT:PSS (PH1000) thin films were prepared by using four different concentrations of DMSO. The concentration was varied in the PEDOT:PSS (PH1000):DMSO solution and the samples were compared with a pristine sample. For each solution two samples were prepared according to low and high spin coating speeds. This procedure allowed us to obtain samples with controlled thicknesses. The thickness, surface roughness and conductivity values are listed in Table 1. The thinner samples around 50 nm and thicker ones around 120 nm are studied to find out the influence of thickness and roughness on the physical properties. To our surprise, the surface roughness hardly varies by a factor of two by changing the sample preparation conditions. However, the trend indicates that roughness slightly increases upon increasing the concentration of DMSO and also on thicker samples; yet a roughness of 1–3 nm in a sample of 50–120 nm is only in the marginal level. The influence of DMSO on the PEDOT:PSS (PH1000) surface morphology as functions of a concentration as well as a function of thickness is presented in Fig. 2. Layers without and with very low concentration of DMSO shows relatively smoother surface with roughness from granular structure of PEDOT:PSS. With an increasing concentration of DMSO a noticeable increase of the roughness of the films can be observed. This is due to the fact that small grains of the PEDOT:PSS (PH1000) are formed. The optical properties were obtained by fitting spectroscopic ellipsometric measurements in the NIR–VIS–UV range (energies between 0.73 and 6 eV). The complex reflectance ratio $\rho =\frac{r_{p}}{r_{s}}=\tan\psi e^{\mathrm{i\Delta}}$ was measured. In the equation ρ is the ratio of the complex reflection coefficients, r_p_ and r_s_ are the complex Fresnel reflection coefficients for p- and s-polarized light, respectively, tan ψ represents the absolute value of the ratio and Δ describes the phase difference between p- and s-polarized light. The dielectric functions of the PEDOT:PSS were fitted with the known thickness and the assumption of a negligible roughness (i.e. that the roughness is much smaller than the wavelength). For fitting procedures, a Drude-ansatz was used with a generic oscillator. Then, after obtaining a reasonable mean square error, a direct inversion procedure was employed to obtain *ε*_1_(*ω*), *ε*_2_(*ω*). The values are accurate at the same level than the thickness values, which were measured by profiling on 4 spots with an average deviation of 3–5%. All data presented represent the true dielectric function, not the pseudodielectric. The comparison between pristine PEDOT:PSS (PH1000) and with DMSO (10 and 20% v/v) is presented in Fig. 3. The real part of the dielectric function (ε_1_) is plotted as a function of energy. The negative values detected below 2 eV confirm the Drude model and let us assume that the charged carriers, contributing to the Drude term, are highly delocalized. Upon the addition of DMSO the appearance of the new broad peak with a maximum around 1.7 eV indicates a shift in the oscillator strength from the Drude tail towards a DMSO related interband transition, as the conductivity increases. These highly delocalized carriers indicate the possibility of a plasma edge around 1.4 eV for pristine PEDOT:PSS. The shift of the plasma edge with the increase of DMSO concentration to 0.76 eV would on the first sight indicate a reduction of the number of charge carriers. However, what can be seen in the imaginary part (ε_2_) of the dielectric as a function, presented in the inset of the Fig. 3, is that with the increasing concentration of DMSO the absorption broadens, which implies that the damping coefficient in the Drude term increases – not surprisingly – in the mixed phase. The total number of electrons, however increases, as can be immediately determined by the sum rule for the dielectric function [19]. By using a numerical Kramers–Kronig transform we checked that the slight shift in the spectra at 2 eV is consistent with that observed in the real part of the dielectric function. The obtained imaginary and real parts of dielectric function were used to calculate real (Re(RI)) and imaginary part (Im(RI)) of the refractive index. The Re(RI) values plotted as a function of energy for pristine PEDOT:PSS and mixed with 10 and 20% v/v DMSO is presented in Fig. 4A. For all samples it can be noticed that Re(RI) value above 3 eV is constant (≈ 1) and independent from solution composition. Below 3 eV for pristine polymer a broad negative peak is observed with a minimum of 0.65 at 1.4 eV. With an increasing concentration of DMSO this negative peak is suppressed and at high concentration it evolves to form a broad positive peak with maximum 1.1 at 1.7 eV. The Im(RI) values for the thin film plotted as a function of energy are presented in Fig. 4B. As expected the shape of plot describing the Im(RI) as a function of energy is similar to the imaginary part of dielectric function. Additionally, similar to the Re(RI) is nearly zero for energies above 3 eV and is independent of layer composition. For pristine PEDOT:PSS the Im(RI) value is almost zero up to 2.4 eV, and then it increases rapidly towards lower energies. At an energy of 0.73 eV the Im(RI) reaches 1.52. With the addition of DMSO the increase of imaginary part of the refractive index sets in at even higher energies (≈ 3 eV) with a smaller increase into the visible and infrared, as compared to the pristine polymer.

**Fig. 5:**
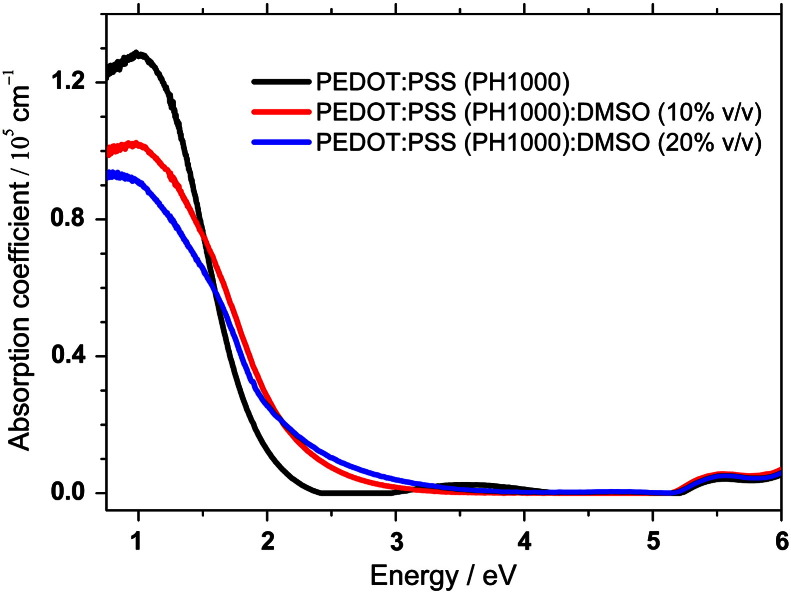
Absorption spectra evaluated from ellipsometric measurement of the pristine PEDOT:PSS (PH1000) (black) and PEDOT:PSS (PH1000) mixed with 10% (red) and 20% (blue) v/v DMSO.

In addition to real and imaginary part of the refractive index, the absorption coefficient (α) was calculated using real and imaginary parts of dielectric function. The results are presented in Fig. 5. PEDOT:PSS has very small absorption coefficient in UV and Vis region, however it increases in the NIR part of the spectrum. With the addition of DMSO there is a noticeable decrease of absorption in the NIR and increase in the visible region. However beside the increase of conductivity, the decrease of the transmission in the visible region is also very important information since PEDOT:PSS is widely used in the optoelectronic devices such as organic solar cells or organic light emitting diodes for which transparency of the electrode is one of the crucial factors in the fabrication of efficiently working device. This shows that for the application of the highly doped PEDOT:PSS as an electrode it is important to balance its electrical and optical properties.

**Fig. 6:**
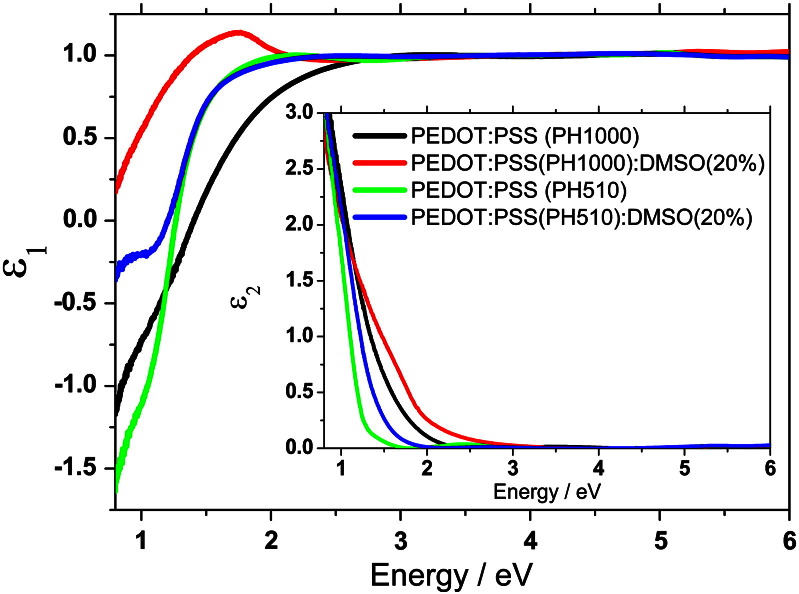
Comparison of the real parts of dielectric function of PEDOT:PSS (PH1000) (black) and PEDOT:PSS (PH1000):DMSO (20% v/v) (red) with lower conducting PEDOT:PSS (PH510) (green) and PEDOT:PSS (PH510):DMSO (20% v/v) (blue).

Furthermore optical properties of PH1000 are compared with a less conducting form of PEDOT:PSS (PH510). The optical and electrical properties of PH510 have already been reported [9]. Although both of these forms of PEDOT:PSS are highly conducting, an interesting observation in the ellipsometric data is shown in the Fig. 6. It is quite evident from the negative values of the real part of the dielectric function below 2 eV for both types of the polymer, prepared with and without DMSO. However a subtle difference in the data below 2 eV is clearly evident. For the pristine PH510 the negative value of the ε_1_ appears at a lower value of 1.2 eV, as compared to PH1000 at 1.5 eV, with significant variation in the shape of the lines. After mixing with DMSO (20% v/v) in PH510 hardly any noticeable variation in the data till 1.3 eV can be observed. However below 1.3 eV the appearance of the new shoulder is related to an interband transition, induced by the presence of DMSO. This is even seen more strongly in the red curve, where a peak in *ε*_1_ is followed by a shoulder in *ε*_2_. The cause for the appearance of the shoulder is the strong varying background due to the metallic dispersion, and this is also consistent, resp. required, by Kramers–Kronig relations. It is interesting that this shift in the oscillator strength is toward lower energies opposite to that observed in the case of PH1000. The prominent peak in the case of PH1000 at 1.7 eV is absent in PH510. Additionally, the comparison of the imaginary part of the dielectric function for PH1000 and PH510 PEDOT:PSS with and without DMSO is presented in the inset of Fig. 6. As it can be noticed for PH510 the value of ε_2_ is close to zero and it strongly increases below 1.5 eV. The addition of DMSO shifts this increase towards higher energies. Nevertheless even for 20% of DMSO in the (PH510): the increase is much lower than for pristine PH1000 mixed with DMSO. This is expected since the first moment of the imaginary part of dielectric function is proportional to the density of the free charges $\left(N\propto \int_{0}^{\infty }\omega \;\varepsilon_{2}\left(\omega \right)\mathrm{d\omega}\right)$. How these variations due to the delocalization of the charge carriers in various types of PEDOT:PSS affect the optical properties has to be investigated in detail in future. The variation of the optical properties for PH1000 has been checked with the four probe conductivity measurements. The conductivity values are presented in Table 1. It was observed that by optimizing the processing condition it is possible to achieve conductivity as high as 1000 S cm^− 1^ in these spin coated samples. The average values of conductivity in these samples tend to be in the range 400–800 S cm^− 1^ upon the addition of DMSO, and in thinner samples conductivity is found be on the lower range. This suggests that the surface roughness affects the conductivity of thinner samples more than the thicker ones. The concentration of DMSO and spinning speed affects the nanomorphology in thinner samples that in turn influences the bulk transport and conductivity. Possibly the chain extension upon the addition of DMSO is correlated to nanomorphology and charge transport properties. As the electronic states get extended the mean free path and conductivity show more metallic-like properties.

## 4 Conclusion

The PEDOT:PSS (PH1000) samples with DMSO concentrations around 10–15% and thickness of 50–100 nm are found to be highly conductive with good optical properties. The spectroscopic ellipsometric characterization of the optical properties is found to be consistent with the conductivity values. These values show that PEDOT based conducting polymers have a good potential to substitute ITO in organic optoelectronic applications.

## Figures and Tables

**Table 1:** The measured root mean square (RMS/nm) roughness and conductivity (σ/S cm^− 1^) values of the different PEDOT:PSS and PEDOT:PSS:DMSO layers.

PEDOT:PSS composition			RMS roughness	σ/S cm^− 1^
Pure PEDOT:PSS	A	45 nm	1.49 nm	0.549
	B	115 nm	1.44 nm	0.689
PEDOT:PSS:DMSO (5% v/v)	C	50 nm	1.64 nm	454
	D	120 nm	1.94 nm	563
PEDOT:PSS:DMSO (10% v/v)	E	50 nm	1.97 nm	402
	F	100 nm	2.43 nm	966
PEDOT:PSS:DMSO (15% v/v)	G	40 nm	1.87 nm	531
	H	120 nm	2.32 nm	866
PEDOT:PSS:DMSO (20% v/v)	I	40 nm	2.25 nm	575
	J	90 nm	2.72 nm	732
